# Autoantigen profiling reveals a shared post-COVID signature in fully recovered and long COVID patients

**DOI:** 10.1172/jci.insight.169515

**Published:** 2023-06-08

**Authors:** Aaron Bodansky, Chung-Yu Wang, Aditi Saxena, Anthea Mitchell, Andrew F. Kung, Saki Takahashi, Khamal Anglin, Beatrice Huang, Rebecca Hoh, Scott Lu, Sarah A. Goldberg, Justin Romero, Brandon Tran, Raushun Kirtikar, Halle Grebe, Matthew So, Bryan Greenhouse, Matthew S. Durstenfeld, Priscilla Y. Hsue, Joanna Hellmuth, J. Daniel Kelly, Jeffrey N. Martin, Mark S. Anderson, Steven G. Deeks, Timothy J. Henrich, Joseph L. DeRisi, Michael J. Peluso

**Affiliations:** 1 Division of Pediatric Critical Care Medicine, UCSF, San Francisco, California, USA.; 2 Chan Zuckerberg Biohub Network, San Francisco, California, USA.; 3 Department of Biochemistry and Biophysics;; 4 Division of HIV, Infectious Diseases, and Global Medicine, Department of Medicine;; 5 Department of Epidemiology and Biostatistics;; 6 Division of Cardiology, Department of Medicine;; 7 Department of Neurology;; 8 Diabetes Center;; 9 Department of Medicine; and; 10 Division of Experimental Medicine, Department of Medicine, UCSF, San Francisco, California, USA.

**Keywords:** COVID-19, Autoimmune diseases

## Abstract

Some individuals do not return to baseline health following SARS-CoV-2 infection, leading to a condition known as long COVID. The underlying pathophysiology of long COVID remains unknown. Given that autoantibodies have been found to play a role in severity of SARS-CoV-2 infection and certain other post-COVID sequelae, their potential role in long COVID is important to investigate. Here, we apply a well-established, unbiased, proteome-wide autoantibody detection technology (T7 phage-display assay with immunoprecipitation and next-generation sequencing, PhIP-Seq) to a robustly phenotyped cohort of 121 individuals with long COVID, 64 individuals with prior COVID-19 who reported full recovery, and 57 pre-COVID controls. While a distinct autoreactive signature was detected that separated individuals with prior SARS-CoV-2 infection from those never exposed to SARS-CoV-2, we did not detect patterns of autoreactivity that separated individuals with long COVID from individuals fully recovered from COVID-19. These data suggest that there are robust alterations in autoreactive antibody profiles due to infection; however, no association of autoreactive antibodies and long COVID was apparent by this assay.

## Introduction

Some individuals do not fully return to baseline health following SARS-CoV-2 infection and experience ongoing morbidity following the acute phase of COVID-19 (1, 2). There is now intense interest in determining the underlying mechanisms of long COVID, one type of post-acute sequelae of SARS-CoV-2 infection (PASC) characterized by symptoms that develop or worsen following COVID-19 that cannot be clearly attributed to another cause (3). Immune dysregulation, including the generation of antibodies against self-antigens, has been suggested as one potential driver of long COVID that warrants further investigation (4, 5).

Acute SARS-CoV-2 infection is associated with the generation of autoreactive antibodies, particularly among individuals with severe disease requiring hospitalization (6–8). For example, one study of 147 individuals hospitalized with COVID-19 found that autoantibodies associated with connective tissue diseases and anti-cytokine antibodies were identified in 50% of samples and tracked with the humoral response to SARS-CoV-2 infection (7). Furthermore, multiple studies have described the contribution of likely preexisting anti-interferon antibodies to vaccine breakthrough infections and severe manifestations of COVID-19, including death (9–12).

Although much work has been done exploring the potential virologic and immunologic factors driving long COVID, the evaluation of autoantibodies in this condition has been more limited. Measurement of anti-nuclear antibodies (ANAs) on standard clinical tests has yielded mixed findings, with some studies identifying a high prevalence of ANAs among those with long COVID (13–15) and other studies finding low prevalence consistent with ANA positivity in the general population (16–18). Although perturbations in interferon signaling pathways have been suggested as one potential mechanism of long COVID (4, 15), anti-interferon antibodies have not been identified in most individuals outside of those who had severe acute infection (16). This is consistent with other PASC, such as multisystem inflammatory syndrome in children, which also has no association with anti-interferon antibodies (19).

The identification of previously reported autoantibodies can be performed using targeted assays. However, identifying the full range of novel autoreactive antibodies, whether they are pathologic or not, requires technologies capable of high-throughput, unbiased, proteome-wide screens. In this study, we screened a cohort of individuals with prior SARS-CoV-2 infection, many of whom met clinical criteria for long COVID, to determine whether a consistent pattern of autoreactivity could be identified. This same technology has been previously utilized to discover novel autoantibodies in a wide range of disease contexts (20–23).

## Results

### Distinct set of autoreactive antibodies in individuals with prior SARS-CoV-2 infection.

**Figure 1:**
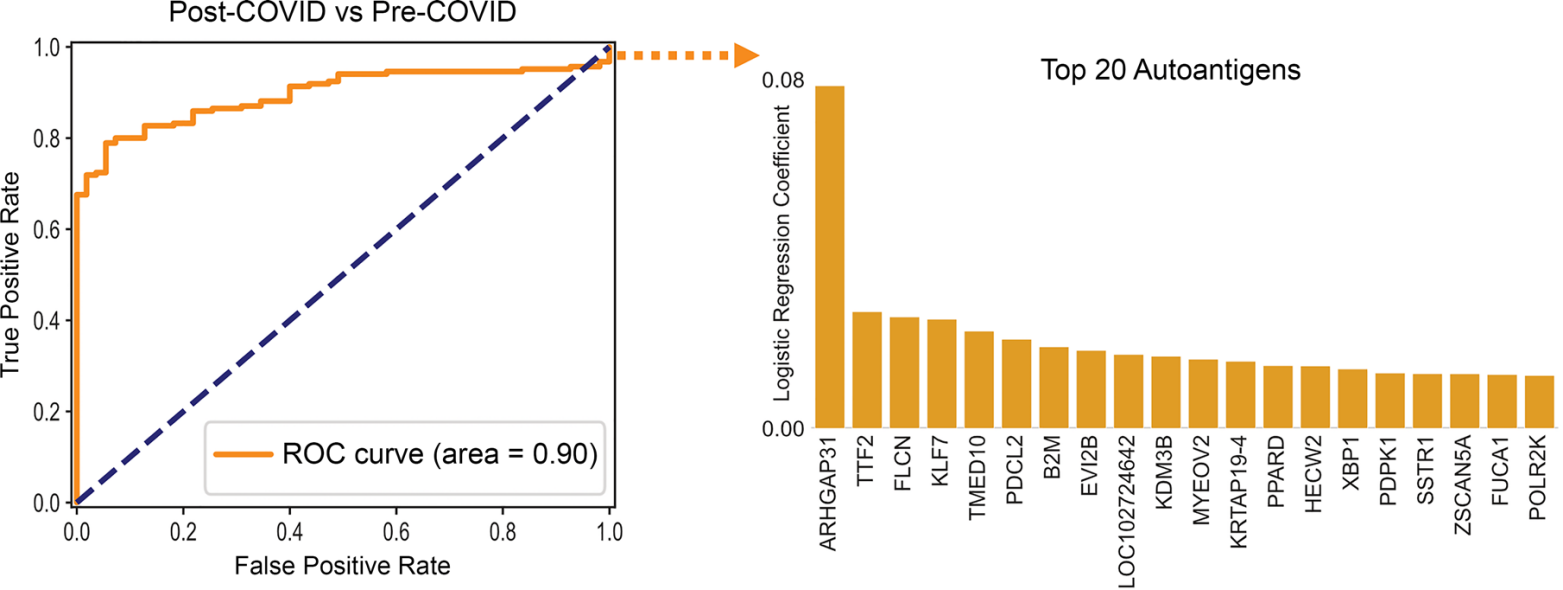
PhIP-Seq autoreactivities distinguish post-COVID sera from pre-COVID controls. Logistic regression comparing PhIP-Seq autoreactivities in all individuals with prior COVID infection compared with pre-COVID controls. Bar plot showing autoreactivities with the top 20 logistic regression coefficients. ROC, receiver operating characteristic.

**Figure 2:**
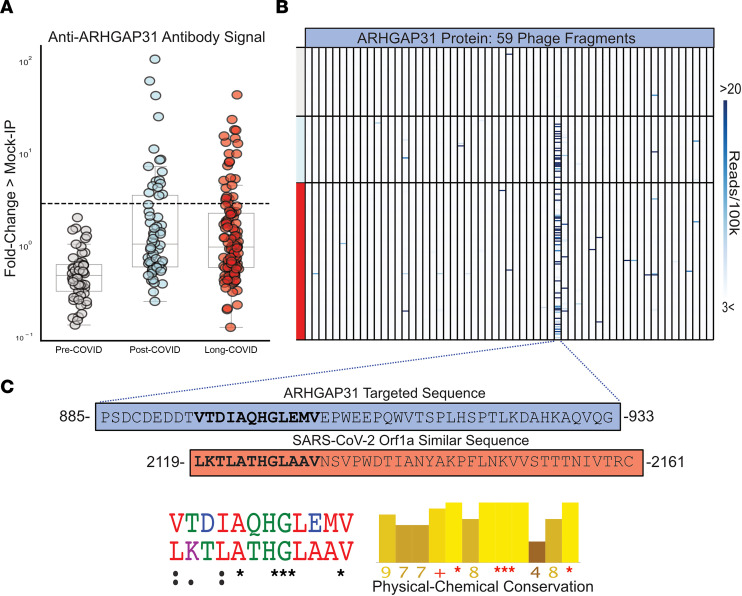
Post-COVID anti-ARHGAP31 autoreactivities target a specific region with similarity to SARS-CoV-2. (**A**) Strip plots showing distribution of ARHGAP31 autoreactivities in long COVID, individuals with prior COVID infection but without long COVID, and pre-COVID controls. Dotted line at 6 standard deviations above mean of pre-COVID controls (underlying box plots showing median, upper and lower quartiles, and whiskers representing 1.5 times the upper and lower interquartile range). (**B**) Distribution of anti-ARHGAP31 autoreactivity signal within ARHGAP31 full-length protein. One specific fragment is targeted. (**C**) Amino acid sequence of the autoreactive region of ARHGAP31 and amino acid sequence of a region of SARS-CoV-2 Orf1a with similarity. Shown below is the multiple sequence alignment (ClustalOmega; asterisk = identical amino acid; colon = strongly similar properties with Gonnet PAM 250 matrix score > 0.5; period = weakly similar with Gonnet PAM 250 matrix score between 0 and 0.5) and strong physical-chemical conservation (Jalview; amino acid physical-chemical conservation scored on a scale of 1–11, asterisk = score of 11 and identical amino acid, plus = 10, all properties conserved).

We employed a previously published proteome-wide approach using a T7 phage-display assay with immunoprecipitation and next-generation sequencing (PhIP-Seq) (20–24). We tested sera from 185 individuals with prior SARS-CoV-2 infection in parallel to sera from 57 individuals collected prior to the known existence of COVID-19 (pre-COVID). Using an unbiased analysis, we identified a distinct pattern of autoreactivity that effectively classified individuals with prior SARS-CoV-2 infection from individuals not yet exposed to the virus with a logistic regression AUC of 0.90 (Figure 1). The protein targets from which these enriched immunoprecipitated peptides were derived are widely varied and lack any apparent shared biological functions or cell type (Figure 1). Among the identified targets, autoreactivity to ARHGAP31 displayed the greatest amount of enrichment, with 22% of individuals with prior SARS-CoV-2 infection yielding enrichment greater than 6 standard deviations of the mean of the pre-COVID controls. No difference in enrichment was observed for those meeting criteria for long COVID with respect to post-COVID comparators (Figure 2A). Additionally, nearly all the autoreactivity was to a single 49–amino acid peptide fragment within the full-length ARHGAP31 protein (Figure 2B), indicating there may be a shared epitope driving this response.

To investigate whether the ARHGAP31 peptide enrichment could be the result of cross-reactivity with antibodies directed against SARS-CoV-2 proteins, we performed a multiple sequence alignment of this 49–amino acid fragment against the full SARS-CoV-2 proteome. A region within the SARS-CoV-2 Orf1a polyprotein was identified with considerable physicochemical similarity to a portion of the autoreactive fragment in ARHGAP31 (Jalview Version 2; Figure 2C), supporting the notion that the observed human peptidome peptide enrichments in post-COVID samples are being driven by anti–SARS-CoV-2 antibodies.

ARHGAP31 is most highly expressed in neuronal cells, Langerhans cells, and endothelial cells (Human Protein Atlas). We were unable to identify any clinical differences between individuals with and without ARHGAP31 autoreactivity, such as differences in frequency of neurologic symptoms.

### Post-COVID autoreactivities are not enriched in individuals with long COVID.

**Figure 3:**
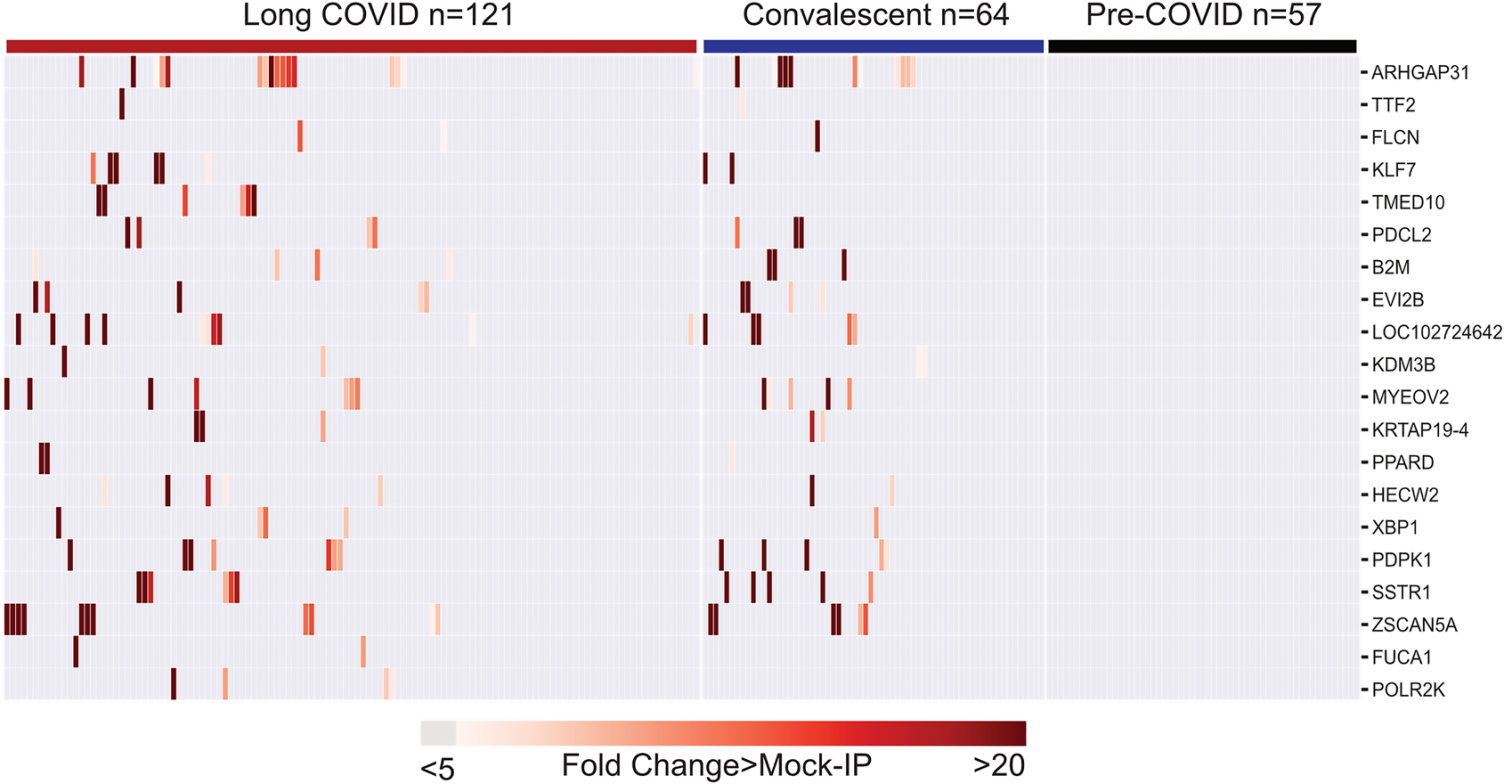
Post-COVID autoreactivities are similarly distributed among long COVID and controls. Hierarchically clustered (Pearson’s) heatmaps showing the PhIP-Seq enrichment for the top 20 autoreactivities ranked by logistic regression coefficient in each patient with long COVID, each convalescent COVID patient, and each pre-COVID control.

To assess whether autoreactive antibodies present in individuals following SARS-CoV-2 infection are associated with long COVID, we compared the distribution of the enriched post-COVID peptides among the 121 individuals with long COVID and the 64 individuals with prior SARS-CoV-2 infection but without long COVID (convalescent COVID). The 20 most enriched proteins with at least 5-fold greater than background (defined as fold-change [FC] over mock IP with protein A/G beads) were compared (Figure 3). Seventeen of the 20 enriched proteins were present in both long COVID and convalescent COVID, and none of these enrichments was observed in any of the pre-COVID controls. Overall, there were no significant differences in enrichment between long COVID and convalescent COVID.

Peptides derived from 3 proteins, TMED10, FUCA1, and POL2RK, were observed only in long COVID; however, the difference did not meet statistical significance. Nonetheless, TMED10 was enriched in 6/121 (5%) of individuals with long COVID. TMED10 is a ubiquitously expressed protein that localizes to the plasma membrane and clusters by single-cell RNA expression most closely with plasma cells and cells involved in the humoral immune response (Human Protein Atlas).

Given that our cohort had more men than women, as well as a substantial number of people living with HIV because of deliberate enrichment (see Methods), we also evaluated the distribution of autoantigens among these groups. There were no autoantigens present exclusively in those with HIV infection; however, peptides derived from TTF2, FLCN, TMED10, KDM3B, MYEOV2, PPARD, and POLR2K were present in only HIV-negative individuals (Supplemental Figure 1A; supplemental material available online with this article; https://doi.org/10.1172/jci.insight.169515DS1). Each autoantigen was present in both men and women, with the exception of HECW2, which was present only in men (Supplemental Figure 1B).

Subgroup analyses were performed to determine whether any of these observed autoreactivities were enriched in particular symptom-defined phenotypes of long COVID. These include cardiopulmonary (cough, shortness of breath, chest pain, palpitations, and fainting), central neurologic symptoms (problems with vision, headache, difficulty with concentration or memory, dizziness, and difficulty with balance), any neurologic symptom (problems with vision, headache, difficulty with concentration or memory, dizziness, difficulty with balance, trouble with smell or taste, phantosmia, or paresthesia), gastrointestinal (diarrhea, constipation, nausea, vomiting, loss of appetite, and abdominal pain), musculoskeletal (back pain; muscle pain; pain in the arms, legs, or joints), and upper respiratory (rhinorrhea and sore throat).

**Figure 4:**
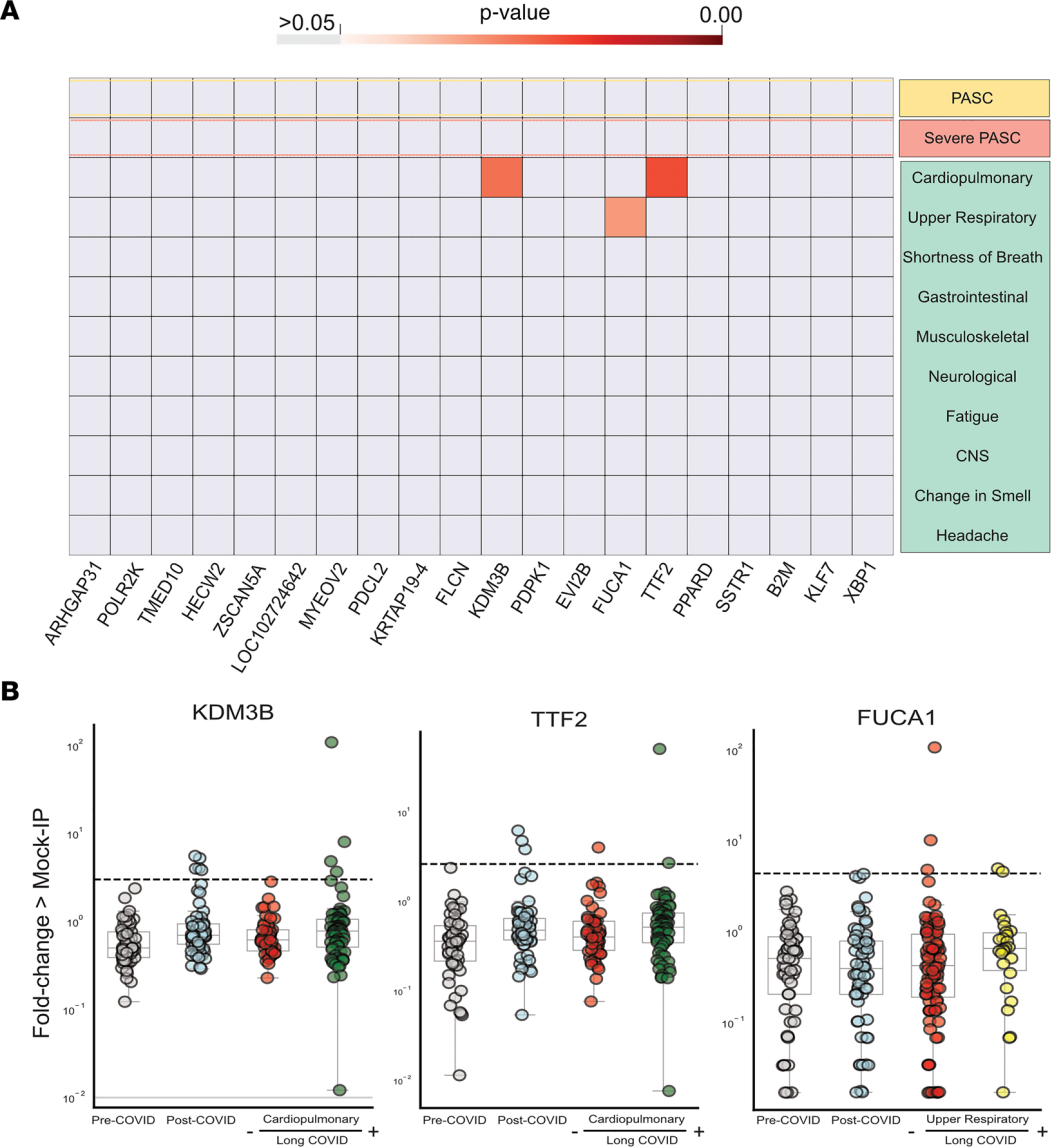
Few significantly increased autoreactivities in long COVID symptom phenotypes. (**A**) Heatmap with *P* values (Kolmogorov-Smirnov testing) of differences in autoantigen enrichment for all individuals with prior COVID infection with and without additional clinical factors. Top row compares those with and without long COVID (long COVID *n* = 121, prior COVID without long COVID *n* = 64). Lower rows show subcategories of long COVID. (**B**) Strip plots showing the 3 autoantibodies with statistically significant enrichment in a post-COVID clinical phenotype (pre-COVID *n* = 57, post-COVID *n* = 64, cardiopulmonary long COVID *n* = 70, upper respiratory long COVID *n* = 26). Dotted lines show 6 standard deviations above the mean of pre-COVID signal (underlying box plots showing median, upper and lower quartiles, and whiskers representing 1.5 times the upper and lower interquartile range).

The peptide enrichments from individuals with particular long COVID phenotypes were compared with the enrichments from individuals with convalescent COVID (no long COVID symptoms) using a 1-sided Kolmogorov-Smirnov test. None of the top 20 autoantibodies were enriched in severe long COVID, and only 3 autoantibodies were statistically increased in any phenotype: TTF2 and KDM3B in those with cardiopulmonary symptoms and FUCA1 in those with upper respiratory symptoms (Figure 4A). However, using a strict cutoff of 6 standard deviations above the pre-COVID controls to determine positivity, none of these autoantibodies was phenotype specific. By looking at the distribution of these antibodies in patients with long COVID with a given phenotype relative to the remaining patients with long COVID and all convalescent COVID patients, it was apparent that the statistical significance in a particular phenotype was driven by either a single individual with extremely high autoantibody signal in the case of KDM3B and TTF2 or higher group signal not meeting the positive threshold cutoff in the case of FUCA1 (Figure 4B). TMED10 autoantigens were not statistically enriched in any particular phenotype.

### Absence of long COVID–specific autoreactivities.

In addition to analyzing the distribution of post-COVID autoreactive peptide enrichments, we also performed additional analyses to detect enrichments that might be present only in long COVID or in particular subcategories of long COVID. To feature-weight enriched peptides, we applied logistic regressions to the degree of enrichment for each individual with long COVID and each individual long COVID symptom phenotype versus enrichment from convalescent COVID patients who did not have the particular symptoms. Given the association between female sex and long COVID, we included women with long COVID as a distinct group in this analysis, in addition to all the previously discussed symptom phenotypes, as well as individuals with difficulty concentrating, worsened quality of life, depression, and generalized anxiety disorder. Women with long COVID and with severe long COVID did not have a distinctive autoantigen profile, with an ROC area under the curve (AUC) of 0.40 and 0.28, respectively (Supplemental Figure 1C). We were similarly unable to identify a set of enriched proteins specific to any of the symptom phenotype subgroups of patients that could effectively distinguish the cohort from controls, as the best ROC AUC was 0.67, and the mean AUC was 0.44 (Supplemental Figure 2).

## Discussion

Autoimmunity has been proposed as one potential mechanism driving long COVID. We applied an unbiased, proteome-wide, validated approach to assess associations between antibody autoreactivity and clinical phenotype. A clear and robust difference in autoreactivity was detected between those infected with SARS-CoV-2 and pre-COVID controls. This difference was constituted by peptides from diverse and varied proteins, most of which are intracellular, suggesting that the origin of the differential enrichment is due to cross-reactivity with SARS-CoV-2–directed antibodies in those who were exposed. A sequence comparison between a peptide from the most enriched protein, ARHGAP31, and Orf1a of SARS-CoV-2 supports this notion, but orthogonal validation through fine-scale epitope mapping and antibody cloning would be required to demonstrate this conclusively. While the clinical significance of incidental autoreactivity due to the humoral immune response to SARS-CoV-2 remains largely unknown, prior studies have identified cross-reactive autoantibodies in severe sequelae of SARS-CoV-2 infection, including in those who develop severe neurological symptoms (25). Understanding the clinical consequences of SARS-CoV-2–driven autoreactivity deserves further attention, perhaps through long-term longitudinal studies.

We found no association to support the hypothesis that autoreactivity, as detected in this assay, contributes to long COVID. Despite numerous successes, PhIP-Seq possesses a number of limitations. Because the T7 phage-displayed peptides are only 49 amino acids, the assay inherently detects mostly linear epitopes. Therefore, complex conformational, posttranslationally modified, or multimeric protein configurations are not predicted to be detectable by this assay, and thus these results do not completely rule out autoimmune interactions in long COVID that are beyond the scope of PhIP-Seq. Furthermore, the study of PhIP-Seq–detected autoantibodies alone does not exclude other forms of autoimmunity in the form of autoreactive T cells or more subtle differences in the autoantibodies themselves with respect to affinity and avidity, which may be distinct in those with and without long COVID. Given recent findings of increased rates of autoimmune conditions following COVID-19 (26), it is also possible that autoreactivity on an individual level may contribute to long COVID, even in the absence of a shared signature.

This analysis has several notable strengths. Participants with SARS-CoV-2 infection were enrolled and assessed prospectively regardless of whether or not they had long COVID symptoms, ensuring that the clinical outcome of interest was measured in a standardized way between the SARS-CoV-2–infected groups. This minimizes the likelihood of confounders that could drive differences between groups if the cohorts had been recruited, assessed, and measured differently. Because the study began enrolling in April 2020, we were able to study participants who had never received a SARS-CoV-2 vaccine prior to biospecimen collection; variability in prior or interval vaccination could potentially confound results of studies like this in cohorts without pre-vaccine samples. In contrast to some early COVID-19 studies, which primarily enrolled individuals hospitalized with severe COVID-19, the cohort was composed primarily of outpatients who had mild-to-moderate SARS-CoV-2 infection, allowing for assessment of long COVID biology in the group representing the vast majority of cases (27). In addition, no participant was known to have experienced a reinfection, and this analysis was performed with specimens from a time when reinfections were uncommon.

Our study also has limitations. First, despite its strengths, the cohort is a convenience sample and not representative of the overall population of people affected by COVID-19. Second, we did not assess the pathophysiology of long COVID among those who had already received a SARS-CoV-2 vaccine, and the biology might differ in this group. Third, our restriction to those who had never received a SARS-CoV-2 vaccine at the time of specimen collection limited our study to those with pre-Delta and pre-Omicron variants, and so additional evaluation of autoreactivity following infection with more modern variants may be warranted. Fourth, people with HIV were overrepresented in our cohort, and although we did not see differential overexpression of autoreactivity in this population, enrichment for this group further limits the representativeness of the cohort. Finally, our classification of long COVID and its symptom phenotypes was based exclusively on participant self-report of symptoms. It is possible that future analyses in more homogenous cohorts, particularly those with objectively measured physiologic perturbations now associated with certain long COVID phenotypes (e.g., postural orthostasis/tachycardia, neurocognitive function deficits, abnormalities on cardiopulmonary exercise testing) may yet reveal a role of autoantibodies in at least a subset of individuals experiencing long COVID. For example, though we were unable to find a clinical association among the 6 individuals with long COVID and enrichment of autoreactivity to TMED10, given that TMED10 is membrane bound (and therefore a potential target for circulating autoantibodies) and highly expressed on immune cells, future study in additional cohorts is warranted.

Long COVID remains a complex clinical entity. Its causes are likely multifactorial, and there is growing consensus that different phenotypes are driven by different pathophysiologic mechanisms (2, 3). Additional work characterizing SARS-CoV-2–specific and autoreactive immune responses in large, well-characterized cohorts over time, during both the acute and post-acute phases of the illness, will be necessary to delineate the biology of long COVID and other PASC and to lead to the development of potential interventions to treat the millions of individuals currently affected by this condition.

## Methods

### Study participants and measurements.

Participants were volunteers in the UCSF Long-term Impact of Infection with Novel Coronavirus (LIINC) study (ClinicalTrials.gov NCT04362150). The details of study design and measurement have been reported previously (28). For the current analysis, we included 185 consecutively enrolled individuals with a history of nucleic acid–confirmed SARS-CoV-2 infection who had a plasma sample collected between 60 and 240 days following initial symptom onset or, if asymptomatic, first positive SARS-CoV-2 test (Supplemental Table 1). Common medical comorbidities include prior history of lung disease (e.g., asthma or bronchitis), autoimmune disease (mainly thyroiditis), diabetes, and hypertension. Notably, the cohort was enriched (*n* = 39, 21%) for people with HIV infection as part of other analyses reported previously (29, 30). Long COVID was defined using study instruments, which have been described in detail elsewhere (28). Briefly, each participant was queried regarding the presence, severity, and duration of 32 physical and mental health symptoms and quality of life. Details of medical history and COVID-19 infection and treatment history were also recorded. Symptoms that predated SARS-CoV-2 infection and that were not changed following infection, as well as those obviously attributed to another cause (e.g., ankle fracture), were not considered to represent long COVID. To avoid confounding by vaccination status and because SARS-CoV-2 vaccines induce divergent B cell responses, all samples included in this study were collected prior to the volunteer ever having received a SARS-CoV-2 vaccine (no participants had a vaccine breakthrough infection or received a SARS-CoV-2 vaccination in the interval between SARS-CoV-2 infection and specimen collection).

Because of challenges in objectively defining long COVID and to thoroughly explore the data in an unbiased manner, we utilized a number of predefined case definitions in our analysis. We constructed case definitions based on symptom presentation and quality-of-life (QOL) responses. Symptom case definitions include the presence of any new or worsening symptoms since SARS-CoV-2 infection (long COVID), presence of 5 or more symptoms (severe long COVID), specific symptom groups according to organ system involvement or phenotypic cluster, and individual symptoms when at least 25 individuals experienced the symptom. For individual and grouped symptom outcomes, we developed 3 potential comparisons between the symptomatic group and (a) all individuals who reported absence of the symptoms of interest, regardless of long COVID status; (b) only individuals who were consistently asymptomatic; and (c) individuals with long COVID but not the symptoms of interest. Severe long COVID was compared only with those without any reported symptoms.

We defined 6 groups of symptoms (symptom phenotypes) based on organ system cluster. These include cardiopulmonary (cough, shortness of breath, chest pain, palpitations, and fainting), CNS-specific (problems with vision, headache, difficulty with concentration or memory, dizziness, and difficulty with balance), any neurologic symptom (problems with vision, headache, difficulty with concentration or memory, dizziness, difficulty with balance, trouble with smell or taste, phantosmia, or paresthesia), gastrointestinal (diarrhea, constipation, nausea, vomiting, loss of appetite, and abdominal pain), musculoskeletal (back pain; muscle pain; pain in the arms, legs, or joints), and upper respiratory (rhinorrhea and sore throat).

QOL was assessed using the EuroQol-5D (EQ-5D), Patient Health Questionnaire depression scale (PHQ-8), and Generalized Anxiety Disorder scale (GAD-7). Individuals with long COVID and the lowest overall QOL score measured via the visual analog scale of the EQ-5D were compared with individuals with the highest overall QOL scores among those with and without long COVID. Individuals with responses categorized as “moderate depression” on the PHQ-8 (score higher than 10) and “moderate anxiety” on the GAD-7 (score higher than 9) were compared with all participants with scores indicating less severe classifications than “moderate depression” and “moderate anxiety” in the following groups: (a) all participants regardless of long COVID status, (b) all participants without long COVID, and (c) all participants with long COVID.

In addition, we recently demonstrated associations between long COVID and other chronic latent viral infections, including serologic evidence suggesting recent Epstein-Barr virus reactivation (30). For this reason, we also used binary variables to create groups indicating the presence of this condition.

### Biospecimen collection.

At each visit, whole blood was collected in EDTA tubes. Plasma was isolated and stored at –80°C until the time of analysis.

### PhIP-Seq.

PhIP-Seq was performed following our previously published vacuum-based PhIP-Seq protocol (23) (https://www.protocols.io/view/scaled-high-throughput-vacuum-phip-protocol-ewov1459kvr2/v1).

### PhIP-Seq analysis.

All analysis (except when specifically stated otherwise) was performed at the gene level, in which all reads for all peptides mapping to the same gene were summed, and 0.5 reads were added to each gene to allow inclusion of genes with 0 reads in mathematical analyses. Within each individual sample, reads were normalized by converting to the percentage of total reads. To normalize each sample against background nonspecific binding, an FC over mock IP was calculated by dividing the sample read percentage for each gene by the mean read percentage of the same gene for the protein A/G bead–only controls. This FC signal was then used for side-by-side comparison between samples and cohorts. Samples that had an FC of 5 or greater were considered enriched for an antibody, and samples with an FC of 6 standard deviations above the mean of pre-COVID controls were considered positive for an autoantibody. FC values were also used to calculate *z* scores for each disease category sample by using each respective control (as specified in figures and Results) and for each control sample by using all remaining controls. These *z* scores were used for the logistic regression feature weighting. In the case of peptide-level analysis, raw reads were normalized by calculating the number of reads per 100,000 reads.

### Statistics.

All statistical analysis was performed in Python using the Scipy Stats package. A 2-way Kolmogorov-Smirnoff test was used for comparisons of FC PhIP-Seq data between groups of samples, except in the case of specifically looking for those genes with increased signal only in the disease cohort, in which a 1-way Kolmogorov-Smirnoff test was employed. The logistic regression machine-learning classifiers were performed using our recently described methods (23). Utilizing the Scikit-learn package, logistic regression classifiers were applied to *z* scored PhIP-Seq values from individuals with a designated disease category versus the designated control. A liblinear solver was used with L1 regularization, and the model was evaluated using a 5-fold cross-validation (4 of the 5 for training, 1 of the 5 for testing).

### Study approval.

Participants provided written informed consent. The study was approved by the UCSF Institutional Review Board.

### Data availability.

Upon publication, all PhIP-Seq data will be made publicly available on https://datadryad.org/: doi:10.7272/Q6Z60M99.

## Author contributions

AB, ST, BG, JDT, JNM, SGD, TJH, JLD, and MJP designed the study. JDK, JNM, SGD, TJH, and MJP designed and oversaw the LIINC cohort, with major contributions from BG, MSD, PYH, and JH. KA, BH, and RH managed cohort operations. Clinical data were collected by JR, BT, RK, HG, and MS. SL and SAG managed the data and developed the clinical data set. ST, SL, SAG, and MJP selected participants for inclusion in this study. Biospecimens were processed in the laboratories of BG and TJH. CYW, AS, and AM performed the measurements in the laboratories of MSA and JLD. AB and AFK performed the data analysis. AB, TJH, JLD, and MJP wrote the first draft of the paper, with input from all other authors. All authors edited the manuscript and approved the final version.

## Supplementary Material

Supplemental data

## Version 1 06/08/2023

Electronic publication

## References

[1] Nalbandian A (2021). Post-acute COVID-19 syndrome. Nat Med.

[2] Davis HE (2023). Long COVID: major findings, mechanisms and recommendations. Nat Rev Microbiol.

[3] Peluso MJ, Deeks SG (2022). Early clues regarding the pathogenesis of long-COVID. Trends Immunol.

[4] Proal AD, VanElzakker MB (2021). Long COVID or post-acute sequelae of COVID-19 (PASC): an overview of biological factors that may contribute to persistent symptoms. Front Microbiol.

[5] Peluso MJ (2022). Long-term immunologic effects of SARS-CoV-2 infection: leveraging translational research methodology to address emerging questions. Transl Res.

[6] Zuo Y (2020). Prothrombotic autoantibodies in serum from patients hospitalized with COVID-19. Sci Transl Med.

[7] Chang SE (2021). New-onset IgG autoantibodies in hospitalized patients with COVID-19. Nat Commun.

[8] Woodruff MC (2022). Dysregulated naive B cells and de novo autoreactivity in severe COVID-19. Nature.

[9] Bastard P (2020). Autoantibodies against type I IFNs in patients with life-threatening COVID-19. Science.

[10] Bastard P (2021). Autoantibodies neutralizing type I IFNs are present in ~4% of uninfected individuals over 70 years old and account for ~20% of COVID-19 deaths. Sci Immunol.

[11] Vazquez SE (2021). Neutralizing autoantibodies to type I interferons in COVID-19 convalescent donor plasma. J Clin Immunol.

[12] van der Wijst MGP (2021). Type I interferon autoantibodies are associated with systemic immune alterations in patients with COVID-19. Sci Transl Med.

[13] Son K (2022). Circulating anti-nuclear autoantibodies in COVID-19 survivors predict long COVID symptoms. Eur Respir J.

[14] Seeßle J (2021). Persistent symptoms in adult patients one year after COVID-19: a prospective cohort study. Clin Infect Dis.

[15] Su Y (2022). Multiple early factors anticipate post-acute COVID-19 sequelae. Cell.

[16] Peluso MJ (2022). Lack of antinuclear antibodies in convalescent coronavirus disease 2019 patients with persistent symptoms. Clin Infect Dis.

[17] Schultheiß C (2022). The IL-1β, IL-6, and TNF cytokine triad is associated with post-acute sequelae of COVID-19. Cell Rep Med.

[19] Bodansky A (2022). NFKB2 haploinsufficiency identified via screening for IFN-α2 autoantibodies in children and adolescents hospitalized with SARS-CoV-2-related complications. J Allergy Clin Immunol.

[20] Mandel-Brehm C (2019). Kelch-like protein 11 antibodies in seminoma-associated paraneoplastic encephalitis. N Engl J Med.

[21] O’Donovan B (2020). High-resolution epitope mapping of anti-Hu and anti-Yo autoimmunity by programmable phage display. Brain Commun.

[22] Vazquez SE (2020). Identification of novel, clinically correlated autoantigens in the monogenic autoimmune syndrome APS1 by proteome-wide PhIP-Seq. Elife.

[23] Vazquez SE (2022). Autoantibody discovery across monogenic, acquired, and COVID-19-associated autoimmunity with scalable PhIP-seq. Elife.

[24] Larman HB (2011). Autoantigen discovery with a synthetic human peptidome. Nat Biotechnol.

[25] Song E (2021). Divergent and self-reactive immune responses in the CNS of COVID-19 patients with neurological symptoms. Cell Rep Med.

[26] Chang R (2023). Risk of autoimmune diseases in patients with COVID-19: a retrospective cohort study. EClinicalMedicine.

[27] Global Burden of Disease Long COVID Collaborators (2022). Estimated global proportions of individuals with persistent fatigue, cognitive, and respiratory symptom clusters following symptomatic COVID-19 in 2020 and 2021. JAMA.

[28] Peluso MJ (2022). Persistence, magnitude, and patterns of postacute symptoms and quality of life following onset of SARS-CoV-2 infection: cohort description and approaches for measurement. Open Forum Infect Dis.

[29] Peluso MJ (2022). Postacute sequelae and adaptive immune responses in people with HIV recovering from SARS-COV-2 infection. AIDS.

[30] Peluso MJ (2023). Chronic viral coinfections differentially affect the likelihood of developing long COVID. J Clin Invest.

