# Supplementary material for: Autoantigen profiling reveals a shared post-COVID signature in fully recovered and long COVID patients
Source: JCI Insight. 2023 Jun 8;8(11):e169515. doi: 10.1172/jci.insight.169515 (PMC10393220; doi:10.1172/jci.insight.169515)
Supplement: Supplemental data [file jciinsight-8-169515-s100.pdf]

	Long COVID (LC) n=121	Convalescent COVID (No LC) n=64	Total n=185
Days post-symptom onset	121 (107 to 129)	121 (108 to 128)	121 (108 to 129)
Number of COVID-attributed symptoms at time of visit	4 (2 to 8)	0 (0 to 0)	2 (0 to 6)
Age in years	46 (38 to 55)	48 (38 to 58)	48 (38 to 56)
Female birth sex, n (%)	57 (47.1)	24 (37.5)	81 (43.8)
Race/ethnicity, n (%)			
Hispanic/Latino	44 (37.0)	12 (19.4)	56 (30.9)
White	61 (51.3)	34 (54.8)	95 (52.5)
Black/African American	6 (5.0)	4 (6.5)	10 (5.5)
Asian	7 (5.9)	9 (14.5)	16 (8.8)
Pacific Islander/Native Hawaiian	1 (0.8)	3 (4.8)	4 (2.2)
Hospitalized during COVID-19 illness, n (%)	30 (24.8)	13 (20.3)	43 (23.2)
Medical Comorbidities, n (%)			
Autoimmune disease	9 (7.4)	1 (1.6)	10 (5.4)
Cancer treated within past 2 years	4 (3.3)	1 (1.6)	5 (2.7)
Diabetes	11 (9.4)	8 (12.5)	19 (10.5)
HIV	31 (25.6)	8 (12.5)	39 (21.1)
Heart attack or heart failure	3 (2.5)	2 (3.1)	5 (2.7)
Hypertension	30 (25.0)	6 (9.4)	36 (19.6)
Lung disease	18 (15.0)	13 (20.3)	31 (16.8)
Kidney disease	1 (0.8)	1 (1.6)	2 (1.1)
History of tobacco smoking, n (%)	37 (32.2)	15 (26.8)	52 (30.4)
Long COVID group*, n (%)		-	-
Any Long COVID	121 (100)	-	-
Severe (5 or more symptoms)	75 (62.0)	-	-
Cardiopulmonary phenotype	70 (57.9)	-	-
Upper respiratory phenotype	26 (21.5)	-	-
Gastrointestinal phenotype	42 (34.7)	-	-
Musculoskeletal phenotype	44 (36.4)	-	-
Neurologic phenotype	100 (82.6)	-	-
Central neurologic (CNS) phenotype	90 (74.4)	-	-
Shortness of breath	44 (36.4)	-	-
Headache	33 (27.3)	-	-
Anosmia	30 (24.8)	-	-
Issues with memory/concentration	63 (52.1)	-	-
Fatigue	59 (48.8)	-	-
Quality of life (low)	49 (40.5)	-	-
Depression (moderate or worse)	26 (21.5)	-	-
Anxiety (moderate or worse)	21 (17.4)	-	-
EBV early antigen-D (EA-D) positive	66 (54.5)	-	-
EBNA high response	77 (63.6)	-	-

Supplemental Table 1. Participant characteristics. All values are median (IQR) unless otherwise noted. *Long COVID phenotypes are not mutually exclusive and include the following: cardiopulmonary (cough, shortness of breath, chest pain, palpitations, fainting), upper respiratory (runny nose, sore throat), gastrointestinal (diarrhea, constipation, nausea, vomiting, loss of appetite, abdominal pain), musculoskeletal (back pain, muscle pain, pain in the arms, legs, or joints), any neurologic symptom (problems with vision, headache, difficulty with concentration or memory, dizziness, difficulty with balance, trouble with smell or taste, phantosmia, paresthesia), CNS-specific (problems with vision, headache, difficulty with concentration or memory, dizziness, difficulty with balance). Counts for common Long COVID symptoms (in which >25 individuals reported the symptom) are shown for shortness of breath, headache, anosmia, and issues with memory/ concentration. Quality of life refers to those with lowest quality-of-life score on a visual analogue scale. Depression counted if the individual scored >10 on PHQ-8. Anxiety counted if the individual scored >9 on GAD-7. As per *Peluso et al J Clin Invest 2023*, EBV early antigen-D positive indicates detectable value above indeterminate range ($\geq$ 9 U/mL) and EBNA high response indicates responses >600 U/mL on the corresponding assay.

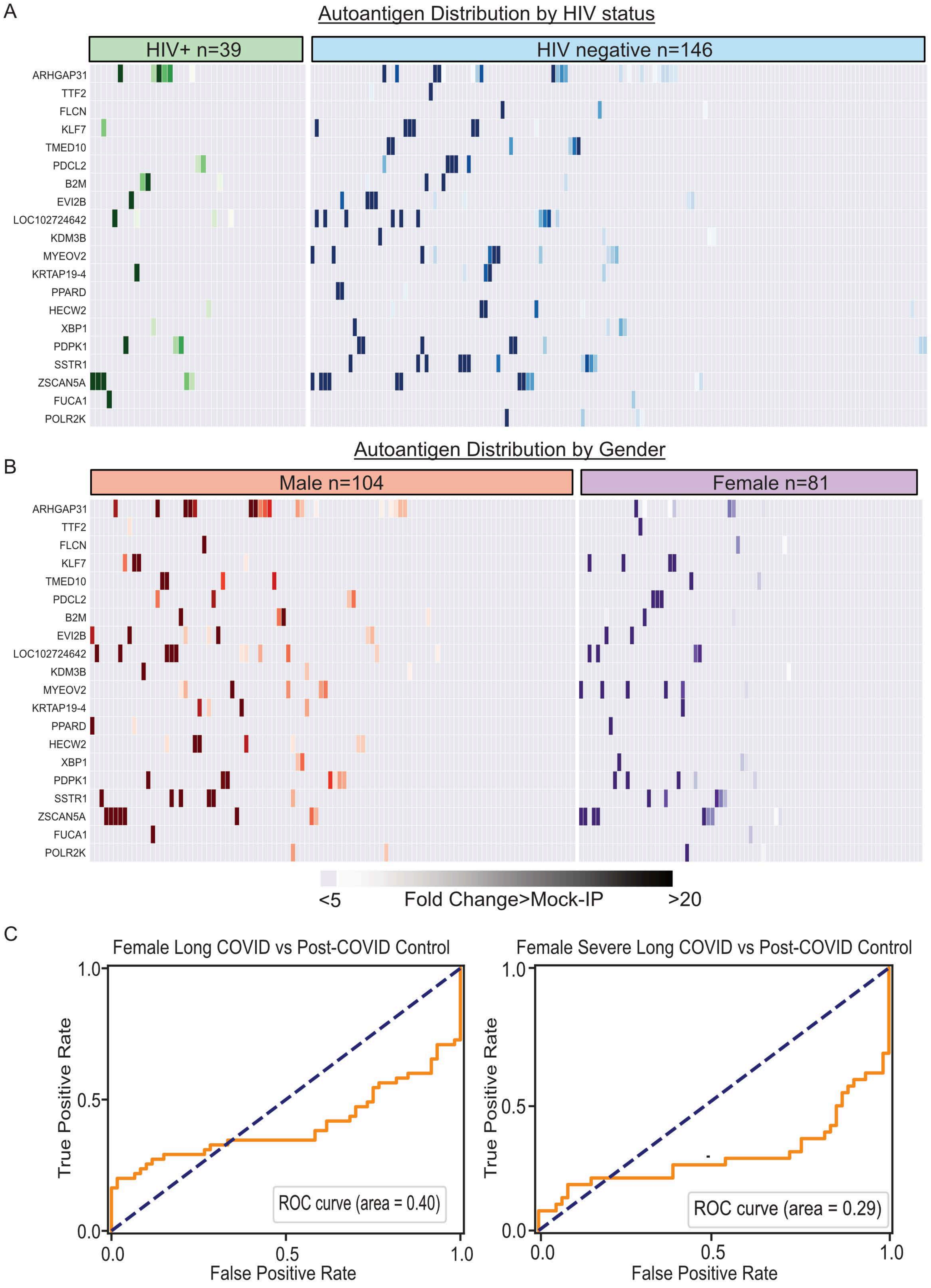
A
Autoantigen Distribution by HIV status
HIV+ n=39
HIV negative n=146
ARHGAP31
TTF2
FLCN
KLF7
TMED10
PDCL2
B2M
EVI2B
LOC102724642
KDM3B
MYEOV2
KRTAP19-4
PPARD
HECW2
XBP1
PDPK1
SSTR1
ZSCAN5A
FUCA1
POLR2K
B
Autoantigen Distribution by Gender
Male n=104
Female n=81
<5
Fold Change>Mock-IP
>20
C
Female Long COVID vs Post-COVID Control
True Positive Rate
False Positive Rate
ROC curve (area = 0.40)
Female Severe Long COVID vs Post-COVID Control
ROC curve (area = 0.29)

Supplemental Figure 1: Distribution of post-COVID autoantigens by HIV status and gender. Hierarchically clustered (Pearson) heatmaps showing the PhIP-Seq enrichment for the top 20 autoreactivities ranked by logistic regression coefficient by HIV status (A; HIV postive n=39, HIV negative n=146) and gender (B; Male n=104, Female n=81).(C) Logistic regression receiver operating characteristic (ROC) curves for female Long COVID patients and female severe Long COVID patients relative to Post-COVID convalescent controls.

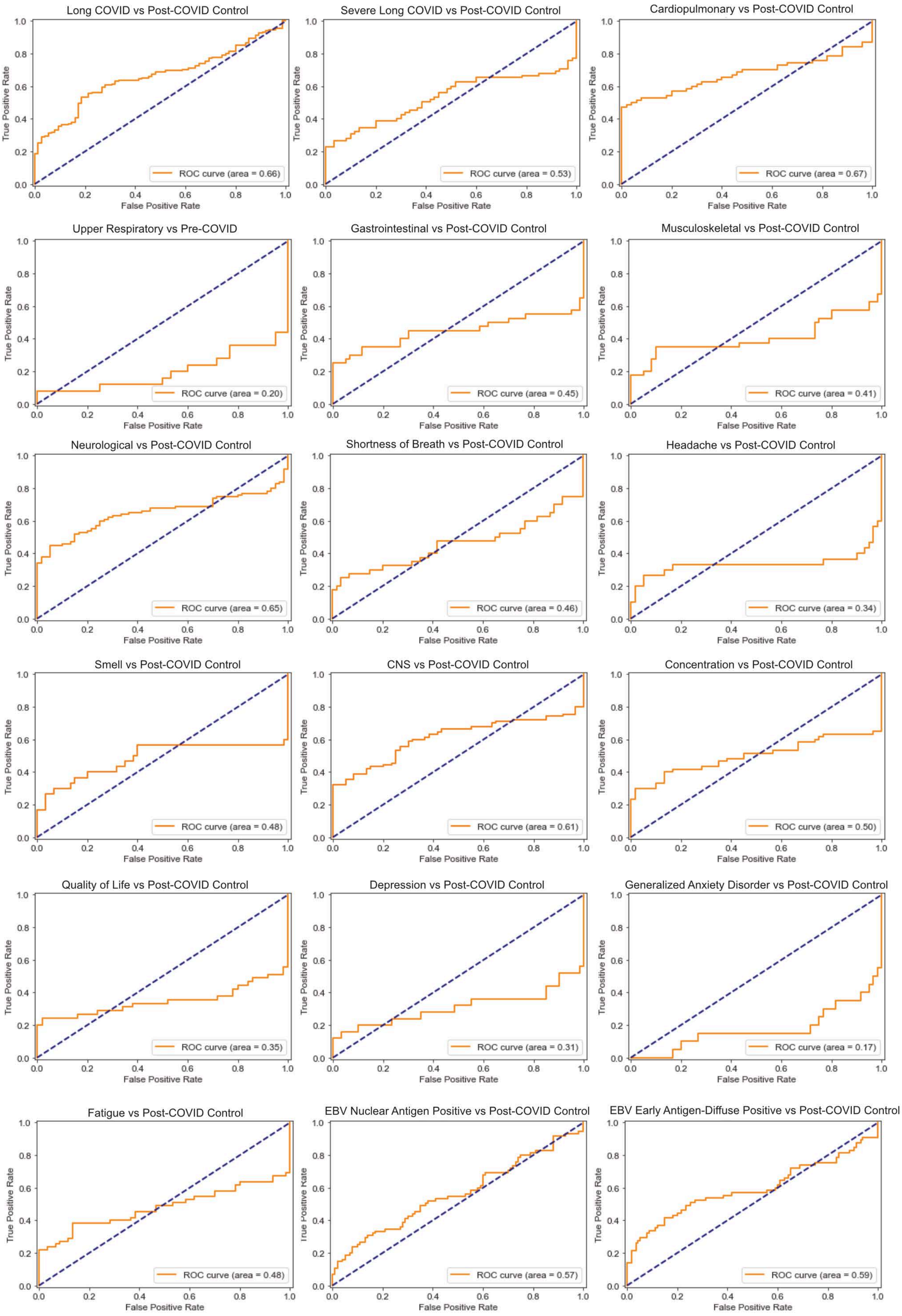
Long COVID vs Post-COVID Control
ROC curve (area = 0.66)
Severe Long COVID vs Post-COVID Control
ROC curve (area = 0.53)
Cardiopulmonary vs Post-COVID Control
ROC curve (area = 0.67)
Upper Respiratory vs Pre-COVID
ROC curve (area = 0.20)
Gastrointestinal vs Post-COVID Control
ROC curve (area = 0.45)
Musculoskeletal vs Post-COVID Control
ROC curve (area = 0.41)
Neurological vs Post-COVID Control
ROC curve (area = 0.65)
Shortness of Breath vs Post-COVID Control
ROC curve (area = 0.46)
Headache vs Post-COVID Control
ROC curve (area = 0.34)
Smell vs Post-COVID Control
ROC curve (area = 0.48)
CNS vs Post-COVID Control
ROC curve (area = 0.61)
Concentration vs Post-COVID Control
ROC curve (area = 0.50)
Quality of Life vs Post-COVID Control
ROC curve (area = 0.35)
Depression vs Post-COVID Control
ROC curve (area = 0.31)
Generalized Anxiety Disorder vs Post-COVID Control
ROC curve (area = 0.17)
Fatigue vs Post-COVID Control
ROC curve (area = 0.48)
EBV Nuclear Antigen Positive vs Post-COVID Control
ROC curve (area = 0.57)
EBV Early Antigen-Diffuse Positive vs Post-COVID Control
ROC curve (area = 0.59)
True Positive Rate
False Positive Rate

Supplemental Figure 2: PhIP-Seq is unable to distinguish Long COVID symptom phenotypes from controls. Logistic regression receiver operating characteristic (ROC) curves for Long COVID patients with different specified symptom phenotypes relative to patients previously infected with COVID without the phenotype. Control refers to post-COVID comparator without the specific disease phenotype indicated in the panel.
